# Safety and T Cell Modulating Effects of High Dose Vitamin D_3_ Supplementation in Multiple Sclerosis

**DOI:** 10.1371/journal.pone.0015235

**Published:** 2010-12-13

**Authors:** Joost Smolders, Evelyn Peelen, Mariëlle Thewissen, Jan Willem Cohen Tervaert, Paul Menheere, Raymond Hupperts, Jan Damoiseaux

**Affiliations:** 1 School for Mental Health and Neuroscience, Maastricht University Medical Center, Maastricht, The Netherlands; 2 Division of Clinical and Experimental Immunology, Department of Internal Medicine, Maastricht University Medical Center, Maastricht, The Netherlands; 3 Academic MS Center Limburg, Orbis Medical Center, Sittard, The Netherlands; 4 Department of Clinical Chemistry, Maastricht University Medical Center, Maastricht, The Netherlands; 5 Laboratory for Clinical Immunology, Maastricht University Medical Center, Maastricht, The Netherlands; New York University, United States of America

## Abstract

**Background:**

A poor vitamin D status has been associated with a high disease activity of multiple sclerosis (MS). Recently, we described associations between vitamin D status and peripheral T cell characteristics in relapsing remitting MS (RRMS) patients. In the present study, we studied the effects of high dose vitamin D_3_ supplementation on safety and T cell related outcome measures.

**Methodology/Principal Findings:**

Fifteen RRMS patients were supplemented with 20 000 IU/d vitamin D_3_ for 12 weeks. Vitamin D and calcium metabolism were carefully monitored, and T cell characteristics were studied by flowcytometry. All patients finished the protocol without side-effects, hypercalcaemia, or hypercalciuria. The median vitamin D status increased from 50 nmol/L (31–175) at week 0 to 380 nmol/L (151–535) at week 12 (P<0.001). During the study, 1 patient experienced an exacerbation of MS and was censored from the T cell analysis. The proportions of (naïve and memory) CD4^+^ Tregs remained unaffected. Although Treg suppressive function improved in several subjects, this effect was not significant in the total cohort (P = 0.143). An increased proportion of IL-10^+^ CD4^+^ T cells was found after supplementation (P = 0.021). Additionally, a decrease of the ratio between IFN-γ^+^ and IL-4^+^ CD4^+^ T cells was observed (P = 0.035).

**Conclusion/Significance:**

Twelve week supplementation of high dose vitamin D_3_ in RRMS patients was well tolerated and did not induce decompensation of calcium metabolism. The skewing towards an anti-inflammatory cytokine profile supports the evidence on vitamin D as an immune-modulator, and may be used as outcome measure for upcoming randomized placebo-controlled trials.

**Trial Registration:**

Clinicaltrials.gov NCT00940719

## Introduction

Multiple Sclerosis (MS) is an inflammatory disease of the central nervous system, probably of autoimmune origin. It is presumed to arise from a combination of genetic and environmental factors [1]. One of the environmental factors which gained much attention during the last decades, is vitamin D. A poor exposure to either vitamin D or sunlight, its most important inducer, has been associated with a high risk of developing MS [2]. The underlying mechanism has not been unravelled yet, but a central role for the actions of vitamin D on immune regulation has been proposed [3], [4]. The biologically active metabolite of vitamin D, 1,25-dihydroxyvitamin D (1,25(OH)_2_D), has potent immune modulating properties, both *in vitro* and in the experimental animal model of MS, Experimental Autoimmune Encephalomyelitis (EAE) [5], [6]. *In vitro*, exposure to 1,25(OH)_2_D inhibits CD4^+^ T cell proliferation and pro-inflammatory cytokine production (IFN-γ and IL-17), and promotes anti-inflammatory cytokine production (IL-4 and IL-10) and acquisition of regulatory T cell (Treg) phenotype [4].

Not only incidence, but also disease activity of MS has been associated with vitamin D status. Disability of MS is inversely correlated with vitamin D status [7], [8], but the causality of this association is uncertain [9]. Additionally, a poor vitamin D status has been associated with an increased risk on relapses [7], [10], [11]. A recent study suggested an increased proportion of relapse free relapsing remitting MS (RRMS) patients in a cohort supplemented with ≈10 000 IU vitamin D_3_/d for 52 weeks [12]. The immune modulating properties of vitamin D have been proposed to underlie these associations. Supplementation of 1 000 IU vitamin D_3_/d for 6 months induced elevated circulating TGF-β levels in MS patients [13]. We studied the correlation of vitamin D status with T cell regulatory status in a cross-sectional design [14]. Although no correlations with the circulating numbers of CD4^+^ Treg were found, vitamin D status correlated positively with the suppressive capacity of Treg. Additionally, the ratio between pro-inflammatory IFN-γ^+^ and anti-inflammatory IL-4^+^ CD4^+^ T cells was higher in RRMS patients with a poor vitamin D status. These associations were independent from the effects of vitamin D on calcium homeostasis [15].

These clinical observations and experimental data on vitamin D and MS warrant the development of well designed clinical trials to assess the disease and immune modulating effects of vitamin D in MS. The aim of the present study was to explore the safety and the *in vivo* effects on the peripheral T cell compartment of high dose vitamin D*_3_* supplementation in MS.

## Materials and Methods

### Subjects

Between October – December 2009, 15 RRMS patients were enrolled [16]. Inclusion criteria were age >18 years, disease duration <6 years, use of Beta Interferon (IFN-β) 1a or 1b, and being relapse free ≥6 weeks prior to enrolment. Exclusion criteria were current use of drugs associated with an increased susceptibility to hypercalcaemia, treatment with immune modulating/suppressive drugs other than IFN-β ≤6 weeks prior to enrolment, pregnancy, hypercalcaemia, serum creatinine >100 µmol/L, and a history of primary hyperparathyroidism, hypercalcaemia, renal dysfunction, cardiac disease, malignancy, or granulomatous disease. Holidays to sunny locations and the use of solariums were not allowed. Continuation of multivitamins containing vitamin D≤600 IU µg/day was allowed. Written informed consent was obtained from each subject. The regional ethical committee ‘Atrium-Orbis-Zuyd’ and the institutional ‘Advisory Board on Scientific Research’ approved this study. This study is registered at www.clinicaltrials.gov as NCT00940719. The protocol for this trial is available as supporting information; see Protocol S1.

### Vitamin D_3_ supplementation

To achieve serum 25-hydroxyvitamin D (25(OH)D) levels comparable to the study by Burton et al. within 12 weeks [12], participants were supplemented with 20 000 IU vitamin D_3_/d. An oil-based vitamin D_3_ solution in a concentration of 20 000 IU/mL was obtained (Vigantol Oil; Merck Serono, Darmstad, Germany) and patients were instructed to ingest 1 mL each morning [17]. No dose-escalation was incorporated, no dietary restrictions were imposed, and no additional calcium supplementation was provided. Vitamin D_3_ solution was provided twice for 6 weeks. At the end of week 6 and 12, residual volume of the vitamin D_3_ solution was assessed. Additionally, patients registered their vitamin D_3_ intake daily on a calendar. As a safety follow-up, a visit was performed at week 16. To further investigate safety, we added an additional visit at week 24 to the protocol.

### Biochemical analysis

At each study visit, calcium, albumin, and 25(OH)D were measured. Additionally, we periodically measured parathyroid hormone (PTH), creatinine, urea, electrolytes and liver function enzymes [alanine transaminase (ALT), aspartate transaminase (AST), and alkaline phosphatase (ALP)]. Additionally to the original protocol, a urine sample was obtained the morning of the study visits on week 12 and 24, to determine the ratio between the molar concentrations of calcium and creatinine. We measured PTH on the Immunolite 2500analyzer (Siemens Healthcare Diagnostics, Deerfield, IL, USA). Other serum and urine biochemical analytes were measured on the Beckman DXC880i analyzer (Beckman Coulter, Woerden, The Netherlands). A radioimmunoassay kit (Immunodiagnostic Systems, Boldon, UK) was used to measure serum 25(OH)D and 1,25(OH)_2_D concentrations. Toxicity of vitamin D_3_ manifests as hypercalcemia (serum total calcium ≥2.6 mmol/L) or hypercalciuria (urinary calcium to creatinine ratio >1.0 mmol/L x (mmol/L)^−1^) [18].

### Cell isolation and purification

Cell isolation was performed as described before [14]. PBMC were isolated by Ficoll gradient centrifugation (Histopaque; Sigma Aldrich, Zwijndrecht, The Netherlands). CD4^+^ T cells were isolated by negative selection with RosetteSep (Stem Cell Technologies, Grenoble, France). CD4^+^ T cells were incubated at 4°C for 30 minutes with anti-CD4-APC (Biolegend, Uithoorn, The Netherlands, #300514), anti-CD25-PE (BD Biosciences, Breda, The Netherlands, #555432) and anti-CD127-FITC (E-Bioscience, Hatfield, UK, #11-1278). Human CD4^+^CD25^+^CD127^−^ Treg [19], and CD4^+^CD25^−^ responder T cells (Tresp) were sorted on a FacsAria (BD Biosciences) cell sorter. After the sort, the median proportion CD4^+^CD25^+^CD127^−^ cells of all events was 98.9% (97.6–99.5) in the Treg tube, and the median proportion CD4^+^CD25^−^ cells was 99.1% (91.0–99.6) in the Tresp tube. Accessory cells were obtained by irradiating autologous PBMC with 66.2 gray.

### Proliferation suppression assay

The CFSE-based proliferation suppression assay was performed as described before [14]. In short, the freshly isolated Tresp were labelled with CFSE (Molecular Probes Invitrogen, Breda, The Netherlands). In a U-bottom 96-wells plate, 2*10^4^ Tresps were stimulated with soluble anti-CD3 (2.0 pg/mL, WT32 IgG2a monoclonal antibody, kindly provided by dr. W Tax, Radboud University Nijmegen Medical Centre, Nijmegen, The Netherlands) in the presence of 1*10^5^ irradiated accessory cells. Tresp were co-cultured for 5 days with varying amounts of Treg (Treg/Tresp ratios 0/1, 0.25/1, 0.5/1, 1/1 or 2/1). All conditions were performed in triplo in a final volume of 200 µL. Control conditions were incorporated to validate the assay, including monocultures of un-stimulated Tresp, and of stimulated accessory cells, Treg, and surpluses of Tresp. After culture, cells were stained with anti-CD4-APC and 7AAD (BD Biosciences, #559925), and the CFSE signal of cells in the CD4^+^7AAD^−^ lymphogate was analysed by flowcytometry on a FACS Canto II flowcytometer (BD Biosciences). Data were analysed with FACS Diva software version 6.1.2. (BD Biosciences). The amount of proliferation achieved in the 0/1 ratio was set at 0% suppression. The median relative amount of suppression in the Treg/Tresp co-cultures was calculated. By linear interpolation, the inhibitor ratio (Treg/Tresp) ratio at which 40% suppression of proliferation was achieved (IR40) was calculated.

### T cell phenotyping and cytokine analysis

Treg were defined as CD25^+^FoxP3^+^ CD4^+^ T cells [20] and as CD25^+^CD127^−^ CD4^+^T cells [19]. Furthermore, CD45RA expression was used to stratify CD25^+^CD127^−^ CD4^+^ T cells for memory (CD45RA^−^) and naïve (CD45RA^+^) Treg [21]. For phenotyping, PBMC were stained with different combinations of anti-CD3-horizon 450 (BD biosciences, #560365), anti-CD3- PerCP (BD biosciences, #27355), anti-CD4-APC, anti-CD4-PerCP (Biolegend, #300528), anti-CD25-PECy7 (BD Biosciences, #54679), anti-CD45RA-PE (BD Biosciences, #07804), anti-CD127-FITC or anti-FoxP3-PE (E-Bioscience, #12-4776), and analysed on a FACS Canto II flowcytometer. The absolute number of lymphocytes was determined with a haematological cell counter (Beckman Coulter).

The cytokine profile of CD4^+^ T cells was determined by assessing the intracellular cytokine pattern of CD3^+^CD8^−^ lymphocytes, which are further referred to as CD4^+^ T cells, by flowcytometry [14]. PBMC were stimulated for 5 hours with calcium ionomycine (Sigma Aldrich), PMA (Sigma Aldrich) and cytokine excretion was blocked with monensin (BD Biosciences). CD69 expression was included to assess the activation status of the cells. Cells were stained intracellularly with anti-IL-4-PE (Biolegend, #500704), anti-IFN-γ-FITC (BD Biosciences, #340449), anti-IL-17A-PerCP-CY5.5 (Biolegend, #512314), anti-IL-10-APC (Biolegend, #501410) and anti-CD69-PE-CY7 (Biolegend, #310912), and extracellularly with anti-CD3-horizon 450 and anti-CD8-APC-H7 (BD Biosciences, #641400). Samples were analysed on a FACS Canto II flowcytometer.

### Statistical analysis

Statistical analysis was conducted with SPSS version 15.0 software (SPSS inc., Chicago IL, USA) and figures were constructed with GraphPad Prism 5 software (GraphPad Software inc., La Jolla CA, USA). The median and corresponding range (min – max) are provided for continuous variables. Differences between two related samples (week 0 and 12) were assessed with the Wilcoxon signed-ranks test, the Man Whitney U test was used for unpaired testing. Paired testing was applied unless indicated otherwise. A p-value <0.05 was considered statistically significant.

## Results

### Adverse events and patient compliance

The characteristics of the 15 participants are shown in Table 1. All patients completed the 12 week supplementation protocol without side effects other than increased flatulence (N = 1). Patients reported some beneficial changes, including decreased fatigue (N = 8), decreased headache (N = 2), decrease in eczematous eruptions (N = 1). During the 12 weeks of supplementation, 1 patient developed a relapse of MS, at week 7 of the protocol, which was confirmed by the treating neurologist. This patient was treated with pulsed prednisolone therapy. During the follow-up period, 1 additional patient suffered from an MS relapse (week 19), resulting in a mean annualized relapse rate of 0.27 (SD 0.70) over 24 weeks in this cohort, compared to 0.80 (SD 0.77) in the year prior to this study (Mann Whitney U test P = 0.014). No further (serious) adverse events were registered. The median total number of missed days of vitamin D_3_ intake was 0/84 days (0–5), according to the patient's diaries. Measurement of residue in the returned vitamin D bottles revealed a median difference of −0.2/42 mL (−8.0–8.0) at week 6, and of +0.4/42 mL (−7.5–5.0) at week 12.

**Table 1:** Patient characteristics.

	Median/Category	Min-Max range/N (%)
Sex	Female	N = 8 (53%)
	Male	N = 7 (47%)
Age	35.15 year	25.47–49.36
MS duration since first symptoms	3.47 year	0.63–5.58
Time since last relapse	0.96 year	0.21–3.35
Number of relapses last 12 months	1.0	0.0–3.0
EDSS score*	2.0	0.5–4.0
Duration of IFN-β use	1.21 year	0.14–4.47
IFN-β type	Avonex®	N = 3 (20%)
	Betaferon®	N = 3 (20%)
	Rebif®	N = 9 (60%)
Race	Caucasian	N = 14 (93%)
	Hispanic	N = 1 (7%)

* Expanded Disability Status Scale [34].

### Biochemical assessment

**Figure 1:**
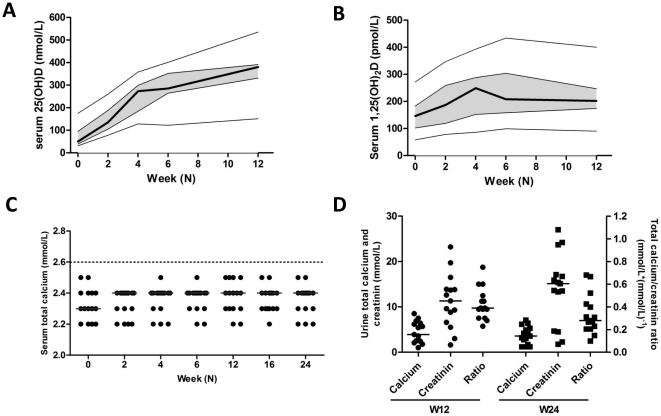
Vitamin D and calcium metabolism during supplementation. A–B) The serum levels of 25(OH)D (A) and 1,25(OH)_2_D (B) are shown for the study visits at week 0, 2, 4, 6, and 12. The thick line indicates the median level, the grey zone the interquartile range. The thin lines show the minimum and maximum values. C) Total serum calcium levels at all study visits (week 0, 2, 4, 6, 12, 16, and 24). Hypercalcaemia is designated as a serum total calcium level ≥2.6 mmol/L. The grey lines indicate the median values. D) Urine levels of total calcium and creatinine levels and their ratio at week 12 and 24. Hypercalciuria is designated as a urine total calcium/creatinine ratio >1.0 mmol/L*(mmol/L)^−1^. The lines indicate the median values.

Serum levels of 25(OH)D increased gradually during the study in all patients (Figure 1A). The median level was 50 nmol/L (31–175) at week 0, and the serum level 25(OH)D of 2 patients exceeded 100 nmol/L (109 and 175 nmol/L, respectively). Remarkably, the patient with the highest serum 25(OH)D level used no vitamin supplements. At week 12, the median 25(OH)D levels was 380 nmol/L (151–535) (P<0.001). Additionally, a rise in serum 1,25(OH)_2_D levels was observed, which stabilized after 4 weeks (Figure 1B). After 6 weeks of supplementation, a decrease in serum PTH was observed (Table 2). Serum uncorrected and albumin corrected calcium levels were not significantly elevated, and remained below 2.6 mmol/L in all subjects (Figure 1C). Additionally, no hypercalciuria (urinary calcium to creatinine ratio >1.0) was observed (Figure 1D). Liver enzyme tests remained unaffected. However, serum creatinine levels gradually increased during vitamin D_3_ supplementation, but remained stable during the follow-up visits (Table 2).

**Table 2:** Biochemical follow-up.

	Supplementation of vitamin D_3_								Follow-up after cessation					
	W0		W6			W12			W16			W24		
Parameter	Median	(Min–Max)	Median	(Min–Max)	P^B^	Median	(Min–Max)	P^B^	Median	(Min–Max)	P^B^	Median	(Min–Max)	P^B^
25-hydroxyvitamin D	50	31–175	285**	122–401	0.001	380**	151–535	0.001	-	-	-	-	-	-
1,25-dihydroxyvitamin D	146	58–272	208**	99–434	0.002	202**	90–400	0.003	-	-	-	-	-	-
Total calcium (mmol/L)	2.3	(2.2–2.5)	2.4	(2.2–2.5)	0.133	2.4	(2.2–2.5)	0.208	2.4	(2.2–2.5)	0.176	2.4	(2.2–2.5)	0.337
Albumin (g/L)	39	(32–47)	39	(31–44)	0.134	39	(31–46)	0.606	39	(31–43)	0.284	38	(31–46)	0.546
Corrected calcium (mmol/L)^A^	2.36	(2.14–2.52)	2.38*	(2.32–2.56)	0.048	2.42	(2.26–2.50)	0.082	2.40	(2.24–2.58)	0.069	2.38	(2.24–2.50)	0.396
Phosphorus (mmol/L)	1.0	(0.7–1.1)	1.1	(0.8–1.4)	0.057	1.1**	(0.9–1.5)	0.008	1.0*	(0.8–1.4)	0.023	1.0	(0.8–1.3)	0.383
PTH (pmol/L)	4.6	(1.9–10.5)	2.8**	(1.7–6.7)	0.003	-	-	-	3.5**	(0.9–9.6)	0.003	3.3*	(0.4–7.5)	0.003
Sodium (mmol/L)	139	(136–142)	139	(138–143)	0.150	141**	(138–143)	0.005	140*	(137–142)	0.048	140	(136–142)	0.080
Potassium (mmol/L)	4.1	(3.4–4.6)	4.0	(3.5–4.6)	0.414	4.5**	(3.6–4.7)	0.008	4.3	(3.6–4.7)	0.555	4.0	(3.7–4.3)	0.133
Urea (mmol/L)	3.5	(2.3–6.0)	3.5	(2.4–6.9)	0.776	3.7	(2.6–6.4)	0.221	3.8	(2.7–5.8)	0.073	3.9	(2.6–6.0)	0.055
Creatinine (µmol/L)	65	(47–84)	69	(49–99)	0.060	73*	(49–109)	0.018	74*	(49–97)	0.012	73	(41–93)	0.064
AST (U/L)	31	(21–42)	32	(21–40)	0.820	-	-	-	29	(20–52)	0.955	28	(19–51)	0.955
ALT (U/L)	34	(19–95)	33	(22–80)	0.975	-	-	-	32	(19–120)	0.670	30	(17–48)	0.900
ALP (U/L)	67	(42–93)	62	(45–85)	0.529	-	-	-	69	(43–85)	0.950	68	(49–99)	0.442

A Calculated with the formula: Calcium_corr_ (mmol/L)  =  total Calcium (mmol/L)+ [(40 – Albumin (g/L))*0.02].

B Difference when compared to baseline levels was tested with the Wilcoxon Signed-Ranks test.

*P<0.050;

**P<0.010.

### Regulatory T cell numbers are not affected by vitamin D supplementation

**Figure 2:**
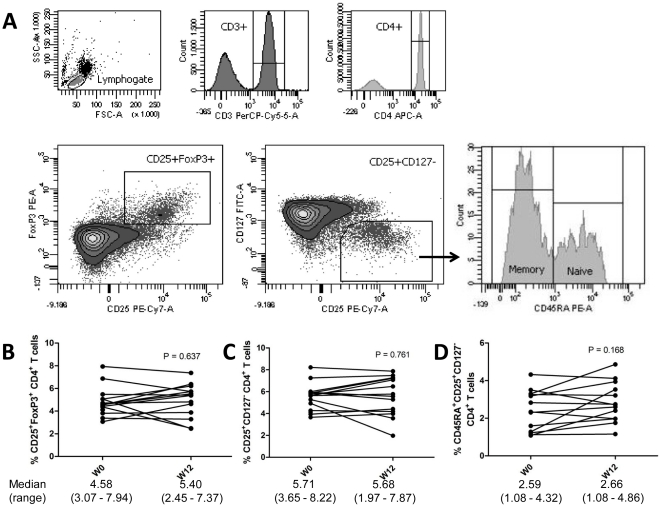
Phenotypic analysis of circulating regulatory T cells (Treg). A) Isolated PBMC were analyzed directly ex-vivo by flow cytometry. In the lymphogate, CD3^+^CD4^+^ cells were assessed for the proportions of CD25^+^FoxP3^+^ and CD25^+^CD127^−^ Treg cells. Expression of CD45RA was analyzed to phenotype naïve (CD45RA^+^) and memory (CD45RA^−^) Treg. B–D) The proportions of circulating CD25^+^FoxP3^+^ (B) and CD25^+^CD127^−^ (C)Treg, and of naïve Treg (D) before and after vitamin D_3_ supplementation (week 0 and 12). Significance was assessed with the Wilcoxon signed ranks comparison test.

The difference in composition and function of the T cell compartment between week 0 and week 12 was assessed in the 14 patients that remained relapse-free during the first 12 weeks. No difference was observed in the absolute amount of T cells in the circulation [1.37×10^6^ (0.91–2.39) vs. 1.32×10^6^ (0.90–2.24) cells/mL; P = 0.761). PBMC were stained directly *ex vivo* to determine the proportions of circulating CD4^+^ Treg cells (Figure 2A). Week 0 and week 12 proportions of CD25^+^FoxP3^+^ and CD25^+^CD127^−^ CD4^+^ Treg did not differ significantly (Figure 2B–C). Proportions of these two Treg phenotypes correlated well (Pearson R = 0.891, P<0.001; ICC = 0.872, P<0.001), and 74.03% (52.09–89.75) of CD25^+^CD127^−^ Treg was FoxP3^+^. The expression of CD45RA was used to stratify Treg for CD45RA^+^ naïve and CD45RA^−^ memory Treg (Figure 2A). There was no difference in the circulating proportions of both naïve and memory Treg between week 0 and week 12 (Figure 2D).

### Modest non-significant improvement of regulatory T cell suppressive function

**Figure 3:**
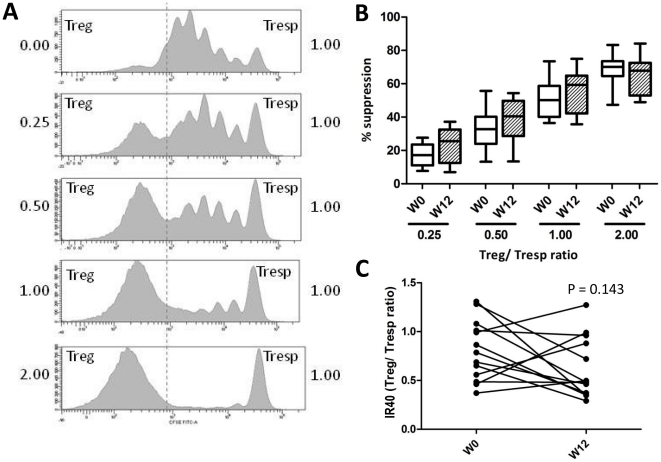
Regulatory T cell suppressive function. A) A representative example of the CFSE signal in the CD4^+^7AAD^−^ lymphogate. The upper panel (0.00) shows proliferation of Tresp cells without Treg. Co-culture with an increasing proportion of Treg results in an inhibition of Tresp cell proliferation (downward panels, ratio 0.25∶1, 0.5∶1, 1∶1 and 2∶1, respectively). An increasing proportion of CFSE^−^ Treg can be observed, and a subsequent decrease of proliferating CFSE^+^ Tresp cells. Between the Treg/Tresp ratio 0.25 and 2.0, all patients achieved at both time points (week 0 and 12) 40% suppression of proliferation (IR40). B) The box and whisker plots show the suppression at each Treg/Tresp ratio before (week 0, open boxes) and after supplementation (week 12, dashed boxes). The boxes show the interquartile range, the whiskers the minimum and maximum value. C) IR40 before and after vitamin D_3_ supplementation (week 0 and 12).

The suppressive capacity of Treg was assessed ex-vivo in a proliferation suppression assay. Of 1 patient, Tresp cells were anergic to anti-CD3 stimulation at week 0. Of the remaining 13 patients, a proliferation suppression assay was acquired at week 0 and week 12. No difference was observed in the amount of Tresp cell proliferation in the culture system [82.8% (53.9–93.3) vs. 83.4% (64.0–96.3); P = 0.848]. At both time points, an increasing suppression of Tresp proliferation was seen with increasing Treg/Tresp ratio (Figure 3). At week 0, the median IR40 was at a Treg/Tresp ratio of 0.79 (0.37–1.31). At week 12, the median IR40 was at 0.48 (0.29–1.27) (Mann Whitney U test P = 0.090). Although Treg suppressive function tended to improve, this improvement was not statistically significant (P = 0.143). Baseline 25(OH)D levels did not differ significantly between improving and non-improving subjects (49 nmol/L (31–109) vs. 74 nmol/L (48–99), respectively; P = 0.260). However, non-improving subjects tended to have better suppression at week 0 when compared to improving subjects (IR40 0.51 (0.37–0.99) vs. 0.86 (0.49–1.31), respectively; P = 0.076).

### CD4^+^ T cell cytokine profile shifts from pro- to anti-inflammatory

**Figure 4:**
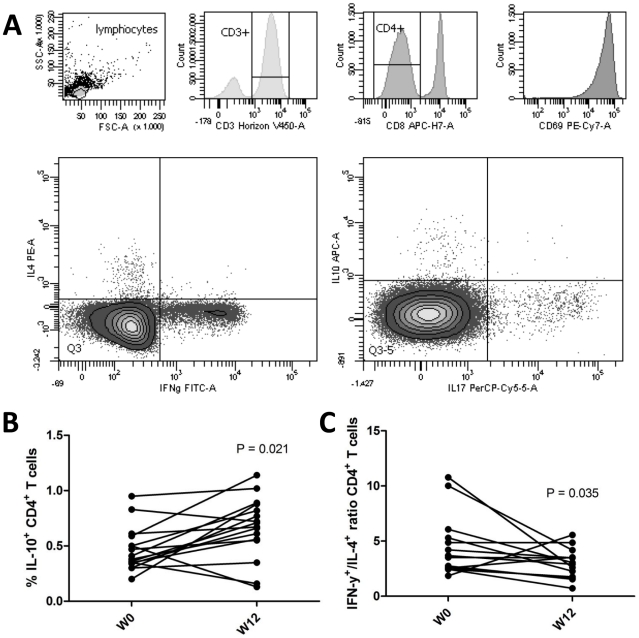
T helper cell cytokine profiles. A) One million PBMC were stimulated for 5 hours with PMA and calcium ionomycin in the presence of monensin, and subsequently stained for flow cytometric analysis. In the lymphogate, CD3^+^CD8^−^ cells were assessed for the proportions of IFN-γ^+^, IL-4^+^, IL-17^+^ and IL-10^+^ cells. CD69 was included to evaluate activation. B) The proportions of IL-10^+^ CD4^+^ T cells before and after supplementation. C) The ratio between Th1 (IFN-γ^+^) and Th2 (IL-4^+^) cells before and after supplementation. Significance was assessed with the Wilcoxon signed ranks comparison test.

The cytokine profile of the CD4^+^ T cells was assessed with an intracellular FACS-staining (Figure 4A). Cytokines of interest were IFN-γ (Th1 cytokine), IL-17 (Th17 cytokine), IL-4 (Th2 cytokine) and IL-10 (regulatory cytokine). The efficacy of stimulation, as evaluated by the expression level of the activation marker CD69, was comparable between week 0 and 12 (median MFI  = 91 030 (45 880–169 520) and 88 580 (56 550–123 710), respectively). When week 0 and 12 were compared, no significant difference was observed in the proportions of CD4^+^ T cells positive for IFN- γ [7.82% (3.45–12.74) vs. 6.51% (1.07–14.66)], IL-17 [0.90% (0.50–2.09) vs. 0.85% (0.16–2.19)], or IL-4 (2.04% (0.80–3.62) vs. 2.37% (0.88–4.21)]. However, the proportion of IL-10^+^ CD4^+^ T cells was significantly increased [0.36% (0.20–0.95) vs. 0.69% (0.13–1.14); P = 0.021] (Figure 4B). When the Th1/Th2 balance was assessed, we found that the IFN-γ^+^/IL-4^+^ balance was decreased after supplementation [3.68 (1.86–10.76) vs. 2.98 (0.71–5.55); P = 0.035] (Figure 4B).

## Discussion

In this study, the effects of high dose vitamin D_3_ supplementation on safety and T cell parameters in patients with RRMS were assessed. We observed that supplementation of 20 000 IU/d vitamin D_3_ for 12 weeks was without negative side-effects and without decompensation of calcium metabolism (i.e. hypercalcaemia and hypercalciuria). The number of Treg remained unaffected, as well as the numbers of Treg memory and naïve subtypes. Results regarding Treg suppressive capacity were inconclusive. However, a shift was observed in the cytokine profiles of CD4^+^T cells, with an increased circulating proportion of IL-10^+^ CD4^+^ T cells, and a decreased ratio of Th1/Th2 cells (IFN-γ^+^/IL-4^+^ CD4^+^ T cell ratio). These results further add to the notion that vitamin D is a physiological immune modulator *in vivo*. Monitoring the cytokine profile of CD4+ T cells could be a valuable tool to assess the immunological efficacy of vitamin D_3_ in randomized controlled clinical trials on vitamin D supplementation in patients with MS.

This study confirmed that supplementation of high doses vitamin D_3_ does not induce a decompensation of calcium metabolism in patients with MS [12], [18]. Without a dose-escalation supplementation scheme, we did not detect hypercalcaemia or hypercalciuria. The slight non-significant increase in serum calcium and phosphorus most likely reflects a correction of vitamin D insufficiency in this cohort. A gradual increase in serum creatinine levels was observed during vitamin D_3_ supplementation, which however remained below 110 µmol/L in all patients. The dramatic complication of a hypercalcaemia associated decrease of glomerular filtration rate (GFR) is unlikely, since neither hypercalcaemia nor hypercalciuria was detected. Earlier dose-escalation trials supplementing up to 280 000 IU/w for 28 weeks and up to 40 000 IU for 52 weeks did not report either significant elevations of serum creatinine levels, or calcifications in the kidneys [12], [18]. Interestingly, however, earlier reports described a rise in serum creatinine which was not accompanied by a decreased GFR in patients treated with 1,25(OH)_2_D or 1α(OH)D [22]. Physiologically, 25(OH)D is filtrated in complex with vitamin D binding protein (DBP) by the glomeruli and re-absorbed by proximal tubular epithelial cells [23]. Interestingly, creatinine is being secreted in the proximal renal tubuli as well. We postulate that a massive increase of 25(OH)D-DBP complex reabsorbtion competes with tubular creatinine excretion and hereby increases serum creatinine levels. Although we believe that the increased serum creatinine levels do not reflect a decreased GFR, future studies on massive dose vitamin D supplementation should be cautious towards patients with an impaired kidney function. On the whole, biochemical data show the absence of vitamin D_3_ toxicity in participants of this study.

We found no effect of vitamin D supplementation on the number of Treg in the circulation. In MS, the function rather than the number of Treg has been reported to be impaired [24], [25], and to relate with serum 25(OH)D levels [14]. Additionally, other immune modulating therapies also have been shown to improve Treg function rather than Treg numbers [26], [27]. An expansion of naïve Treg has been described in Glatiramer Acetate treatment [26], and a decrease of memory Treg cells in IFN-β treatment [27]. Therefore, we also assessed these Treg subsets, but found no effect of vitamin D_3_ supplementation on the proportions of naïve and memory Treg.

Previously, we observed a correlation of Treg suppressive capacity with serum 25(OH)D levels [14]. Although Treg suppressive function was improved in several patients after 12 weeks of vitamin D_3_ supplementation, this improvement was not statistically significant. The size of the cohort assessed is most likely too small to detect a possible effect in this complex assay. Alternatively, one could speculate from our previous work that only in the patients with the poorest vitamin D statuses, an improved suppression can be expected [14]. Although vitamin D statuses at week 0 were lower in the 9 improving patients when compared to the 4 non-improving patients, the difference was not significant. Additionally, Treg suppressive function is not impaired in all RRMS patients, and shows a substantial overlap with healthy individuals [25]–[28]. Therefore, it can be speculated that an adequate Treg function cannot be improved further on vitamin D_3_ supplementation. Indeed, the patients of whom Treg suppressive function improved tended to have a poorer suppression at week 0. Lastly, the genetic background of an individual may affect the immunological response to vitamin D_3_ supplementation [29]. Altogether, the data collected in this study regarding Treg suppressive function remain inconclusive.

A significant effect on the composition of the T helper cell compartment was detected. The ratio of IFN-γ^+^/IL-4^+^ CD4^+^ T cells represents the balance between pro-inflammatory Th1 and anti-inflammatory Th2 cells, and is regarded an important variable in autoimmune diseases [30]. During supplementation, this ratio changed towards a less pro-inflammatory profile. The effect on absolute proportions of IFN-γ^+^ and IL-4^+^ CD4^+^ T cells may be too small to be detected in this small cohort. This is in accordance with previous cross-sectional findings [14]. Interestingly, we detected also more IL-10^+^ CD4^+^ T cells after supplementation. The source of IL10^+^ cells in the CD4^+^ T cell compartment may be the T regulatory cell type 1 (Tr1) [31]. Alternatively, IL-10 has also been proposed to be a typical Th2 cytokine. However, we measured no double producers of IL-10 and the (other) typical Th2 cytokine IL-4 (data not shown). Therefore, we speculate that the rise in IL-10^+^ CD4^+^ T cells might reflect expansion of inducible regulatory Tr1 cells. The finding of an altered profile of pro- and anti-inflammatory CD4^+^ T cells conforms to observations in experimental studies [5] and further supports the assessment of vitamin D_3_ as a natural immune modulator in MS [3].

Although the present study did not comprise a placebo-group, it delivered valuable new information. We earlier performed a cross-sectional study, in which serum 25(OH)D levels correlated with peripheral T cell homeostasis [14]. However, the cross-sectional design did not allow statements on causality, since physical exercise and UV exposure per se could be the real underlying mediators [32]. Our current study revealed 25(OH)D as a good causal candidate. Ultimately, however, placebo controlled studies should demonstrate that vitamin D_3_ is an immune modulator *in vivo*. Regarding these upcoming trials, it is also of interest that we included patients treated with immune modulating drugs (IFN-β). Interestingly, therapy with 1,25(OH)_2_D showed synergistic effects with IFN-β in an EAE model of MS [33]. Upcoming clinical trials will most likely assess add-on effects of vitamin D_3_ on current immune modulating drugs. Results from the present study suggest that it is worthwhile to combine immune-modulating drugs like IFN-β with vitamin D_3_ in order to further modulate the immune system in a for MS beneficial way.

In conclusion, we showed in a cohort of RRMS patients that supplementation of high doses vitamin D_3_ did not result in a decompensated calcium metabolism. Additionally, vitamin D_3_ appeared to skew the CD4^+^ T cell compartment to a more pronounced anti-inflammatory state. Herewith, we confirmed that the peripheral CD4^+^ T cell compartment cytokine profile is a potential outcome measure in upcoming randomized controlled clinical trials. Ultimately, these trials should demonstrate whether vitamin D_3_ is an immune and disease modulating compound in MS.

## Supporting Information

Protocol S1 (DOC)

## References

[1] pston A, Coles A (2008). Multiple Sclerosis.. Lancet.

[2] Ascherio A, Munger KL, Simon C (2010). Vitamin D and multiple sclerosis.. Lancet Neurol.

[3] Hayes CE, Cantorna MT, DeLuca HF (1997). Vitamin D and multiple sclerosis.. Proc Soc Exp Biol Med.

[4] Smolders J, Damoiseaux J, Menheere P, Hupperts R (2008). Vitamin D as an immune modulator in multiple sclerosis, a review.. J Neuroimmunol.

[5] Correale J, Ysrraelit MC, Gaitán MI (2009). Immunomodulatory effects of vitamin D in multiple sclerosis.. Brain.

[6] Cantorna M, Hayes C, DeLuca H (1996). 1,25-dihydroxyvitamin D3 reversibly blocks the progression of relapsing encephalomyelitis, a model of multiple sclerosis.. Proc Natl Acad Sci U S A.

[7] Smolders J, Menheere P, Kessels A, Damoiseaux J, Hupperts R (2008). Association of vitamin D metabolite levels with relapse rate and disability in multiple sclerosis.. Mult Scler.

[8] Van der Mei I, Ponsonby A, Dwyer T, Blizzard L, Taylor BV (2007). Vitamin D levels in people with multiple sclerosis and community controls in Tasmania, Australia.. J Neurol.

[9] Ascherio A, Munger K (2008). Epidemiology of multiple sclerosis: from risk factor to prevention.. Semin Neurol.

[10] Soilu-Hanninen M, Airas L, Mononen I, Heikkila A, Viljanen M (2005). 25-Hydroxyvitamin D levels in serum at the onset of multiple sclerosis.. Mult Scler.

[11] Simpson S, Taylor B, Blizzard L, Ponsonby AL, Pittas F, Tremlett H, Dwyer T, Gies P, van der Mei I (2010). Higher 25-hydroxyvitamin D is associated with lower relapse risk in MS.. Ann Neurol.

[12] Burton JM, Kimball S, Vieth R, Bar-Or A, Dosch HM (2010). A phase I/II dose-escalation trial of vitamin D3 and calcium in multiple sclerosis.. Neurology.

[13] Mahon BD, Gordon SA, Cruz J, Cosman F, Cantorna MT (2003). Cytokine profile in patients with multiple sclerosis following vitamin D supplementation.. J Neuroimmunol.

[14] Smolders J, Thewissen M, Peelen E, Menheere P, Cohen Tervaert JW (2009). Vitamin D status is positively correlated with regulatory T cell function in patients with multiple sclerosis.. PLoS One.

[15] Smolders J, Menheere P, Thewissen M, Peelen E, Cohen Tervaert JW (2010). Regulatory T cell function correlates with serum 25-hydroxyvitamin D, but not with 1,25-dihydroxyvitamin D, parathyroid hormone and calcium levels in patients with relapsing remitting multiple sclerosis.. J Steroid Biochem Mol Biol.

[16] Polman C, Reingold S, Edan G, Filippi M, Hartung HP (2005). Diagnostic criteria for multiple sclerosis: 2005 revisions to the “McDonald Criteria”.. Ann Neurol.

[17] Maalouf J, Nabulsi L, Vieth R, Kimball S, El-Rassi R (2008). Short- and long-term safety of weekly high-dose vitamin D_3_ supplementation in school children.. J Clin Endocrinol Metab.

[18] Kimball SM, Ursell MR, O'Connor P, Vieth R (2007). Safety of vitamin D_3_ in adults with multiple sclerosis.. Am J Clin Nutr.

[19] Liu W, Putnam AL, Xu-Yu Z, Szot GL, Lee MR (2006). CD127 expression inversely correlates with FoxP3 and suppressive function of human CD4+ Treg cells.. J Exp Med.

[20] Fontenot JD, Gavin MA, Rudensky AY (2003). FoxP3 programs the development and function of CD4^+^CD25^+^ regulatory T cells.. Nat Immunol.

[21] Seddiki N, Santner-Nanan B, Tangye SG, Alexander SI, Solomon M (2006). Persistence of naive CD45RA^+^ regulatory T cells in adult life.. Blood.

[22] Andreev E, Koopman M, Arisz L (1992). A rise in plasma creatinine that is not a sign of renal faillure: which drugs can be responsible?. J Int Med.

[23] Doorenbos CRC, van den Born J, Navis G, de Borst MH (2009). Possible renoprotection by vitamin D in chronic renal disease: beyond mineral metabolism.. Nat Rev Nephrol.

[24] Viglietta V, Baecher-Allan C, Weiner H, Hafler D (2004). Loss of functional suppression by CD4^+^CD25^+^ regulatory T cells in patients with multiple sclerosis.. J Exp Med.

[25] Venken K, Hellings N, Hensen K, Rummens JL, Medaer R (2006). Secondary progressive in contrast to relapsing-remitting multiple sclerosis patients show a normal CD4^+^CD25^+^ regulatory T-cell function and FOXP3 expression.. J Neurosci Res.

[26] Haas J, Korporal M, Balint B, Fritzsching B, Schwarz A (2009). Glatiramer acetate improves regulatory T-cell function by expansion of naive CD4^+^CD25^+^FOXP3^+^CD31^+^ T-cells in patients with multiple sclerosis.. J Neuroimmunol.

[27] Korporal M, Haas J, Balint B, Fritzsching B, Schwarz A (2008). Interferon beta-induced restoration of regulatory T-cell function in multiple sclerosis is prompted by an increase in newly generated naive regulatory T cells.. Arch Neurol.

[28] Michel L, Berthelot L, Pettré S, Wiertlewski S, Lefrère F (2008). Patients with relapsing-remitting multiple sclerosis have normal Treg function when cells expressing IL-7 receptor α-chain are excluded from the analysis.. J Clin Invest.

[29] Ramagopalan SV, Maugeri NJ, Handunnetthi L, Lincoln MR, Orton SM (2009). Expression of the multiple sclerosis-associated MHC class II allele HLA-DRB1*1501 is regulated by vitamin D.. PLoS Genet.

[30] Abbas AK, Murphy KM, Sher A Functional diversity of helper T lymphocytes.. Nature.

[31] Moore KW, de Waal Malefyt R, Coffman RL, O'Garra A (2001). Interleukin-10 and the interleukin-10 receptor.. Annu Rev Immunol.

[32] Becklund BR, Severson KS, Vang SV, DeLuca HF (2010). UV radiation suppresses experimental autoimmune encephalomyelitis independent of vitamin D production.. Proc Natl Acad Sci U S A.

[33] van Etten E, Gysemans C, Branisteanu DD, Verstuyf A, Bouillon R (2007). Novel insights in the immune function of the vitamin D system: synergism with interferon-beta.. J Steroid Biochem Mol Biol.

[34] Kurtzke JF (1983). Rating neurologic impairment in multiple sclerosis: an expanded disability status scale (EDSS).. Neurology.

