# Supplementary material for: Safety and T Cell Modulating Effects of High Dose Vitamin D3 Supplementation in Multiple Sclerosis
Source: PLoS One. 2010 Dec 13;5(12):e15235. doi: 10.1371/journal.pone.0015235 (PMC3001453; doi:10.1371/journal.pone.0015235)
Supplement: Protocol S1 — (DOC) [file pone.0015235.s001.doc]

The effects of vitamin D_3 supplementation on the T cell compartment in Multiple Sclerosis; a pilot study

(Version 3, 1-07-2009)

The effects of vitamin D_3 supplementation on the T cell compartment in Multiple Sclerosis; a pilot study

**Protocol ID**	**NL28286.096.09/ 09-T-43**
**Short title**	**Vitamin D_3 supplementation and the T cell compartment in MS**
**Version**	**Version 3**
**Date**	**01-07-2009**
**Coordinating investigator/project leader:**	Prof. dr. R Hupperts, M.D., Ph.D.<br>Neurologist<br>Academic MS Center Limburg/ Department of Neurology<br>Orbis Medical Center, Sittard
**Principal investigator:**	Drs. J Smolders, M.D.<br>PhD-student<br>School for Mental Health and Neuroscience<br>Maastricht University Medical Center (MUMC+)
**Independent physician:**	Dr. P van Paassen<br>Clinical Immunologist/ Nephrologist<br>Department of Internal Medicine<br>Maastricht University Medical Center (MUMC+)
**Collaborators (alphabetical order):**	**Department of Clinical Chemistry**<br>**Maastricht University Medical Center (MUMC+)**<br>*Dr. P Menheere, Clinical Chemist/ Endocrinologist*<br>**Department of Internal Medicine, Division of Clincial and Experimental Immunology**<br>**Maastricht University Medical Center (MUMC+)**<br>*Prof. Dr. JW Cohen Tervaert, Clinical Immunologist*<br>*Dr. J Damoiseaux, Medical Immunologist*

Mw. Dr. M Thewissen Immunologist

Mw. E Peelen, MSc, PhD-student

Laboratory for Hematology and Clinical Chemistry

Orbis Medical Center Sittard

Dr. J ten Kate, Clinical Chemist

Dr. M Vogt, Clinical Chemist

Pharmacy

Orbis Medical Center Sittard

Dr. R Janknegt, pharmacist

PROTOCOL SIGNATURE SHEET

Name	Signature	Date
**Project Leader:**<br><br>***Prof. dr. R Hupperts***<br>***Neurologist***		
**Principal Investigator:**<br><br>***Drs. J Smolders***<br>***PhD-student***		
**Collaborators**<br><br>***Prof. dr. JW Cohen Tervaert***<br>***Immunologist***<br><br>***Dr. J Damoiseaux***<br>***Medical Immunologist***<br><br>***Dr. R Janknegt***<br>***Pharmacist***<br><br>***Dr. P Menheere***<br>***Clinical Chemist/ Endocrinologist***<br><br>***Dr. P van Paassen***<br>***Nephrologist***		

E Peelen, MSc<br>*PhD-student*<br><br>*Dr. J ten Kate*<br>*Clinical Chemist*<br><br>*Dr. M Thewissen*<br>*Immunologist*<br><br>*Dr. M Vogt*<br>*Clinical Chemist*		

TABLE OF CONTENTS

1. INTRODUCTION AND RATIONALE....................11
2. OBJECTIVES....................13
3. STUDY DESIGN....................14
4. STUDY POPULATION....................15
4.1 Population (base)....................15
4.2 Inclusion criteria....................15
4.3 Exclusion criteria....................15
4.4 Sample size calculation....................16
5. TREATMENT OF SUBJECTS....................17
5.1 Investigational product/treatment....................17
5.2 Use of co-intervention....................17
5.3 Escape medication....................17
6. METHODS....................19
6.1 Study parameters/endpoints....................19
6.1.1 Main study parameter/endpoint....................19
6.1.2 Secondary study parameters/endpoints (if applicable)....................19
6.2 Randomisation, blinding and treatment allocation....................19
6.3 Study procedures....................20
6.4 Withdrawal of individual subjects....................21
6.4.1 Specific criteria for withdrawal (if applicable)....................22
6.5 Replacement of individual subjects after withdrawal....................22
6.6 Follow-up of subjects withdrawn from treatment....................22
6.7 Premature termination of the study....................22
7. SAFETY REPORTING....................23
7.1 Section 10 WMO event....................23
7.2 Adverse and serious adverse events....................23
7.2.1 Suspected unexpected serious adverse reactions (SUSAR)....................23
7.2.2 Annual safety report....................24
7.3 Follow-up of adverse events....................24
8. STATISTICAL ANALYSIS....................25
8.1 Descriptive statistics....................25
8.2 Univariate analysis....................25
8.3 Multivariate analysis....................25
9. ETHICAL CONSIDERATIONS....................26
9.1 Regulation statement....................26
9.2 Recruitment and consent....................26
9.3 Objection by minors or incapacitated subjects (if applicable)....................26
9.4 Benefits and risks assessment, group relatedness....................26
9.5 Compensation for injury....................27
9.6 Incentives (if applicable)....................27
10. ADMINISTRATIVE ASPECTS AND PUBLICATION....................28

10.1 Handling and storage of data and documents 28
10.2 Amendments 28
10.3 Annual progress report 28
10.4 End of study report 28
10.5 Public disclosure and publication policy 29

LIST OF ABBREVIATIONS AND RELEVANT DEFINITIONS

1,25(OH)$_2$D	1,25-dihydroxy vitamin D
25(OH)D	25hydroxyvitamin D
ABR	ABR form (General Assessment and Registration form) is the application form that is required for submission to the accredited Ethics Committee (ABR = Algemene Beoordeling en Registratie)
ALT	Alanine aminotransferase
AP	Alkaline phosphatase
AST	Aspartate aminotransferase
AE	Adverse Event
AR	Adverse Reaction
CA	Competent Authority
CCMO	Central Committee on Research Involving Human Subjects
CV	Curriculum Vitae
DSMB	Data Safety Monitoring Board
EU	European Union
EDSS	Expanded Disability Status Scale
EudraCT	European drug regulatory affairs Clinical Trials GCP Good Clinical Practice
IB	Investigator’s Brochure
IC	Informed Consent
IFN-γ	Interferon gamma
IL-4	Interleukin 4
IMP	Investigational Medicinal Product
IMPD	Investigational Medicinal Product Dossier
METC	Medical research ethics committee (MREC); in Dutch: medisch ethische toetsing commissie (METC)
MS	Multiple Sclerosis
PTH	Parathyroid hormone
RRMS	Relapsing Remitting Multiple Sclerosis

(S)AE	Serious Adverse Event
SPC	Summary of Product Characteristics (in Dutch: officiële productinfomatie IB1-tekst)
Sponsor	The sponsor is the party that commissions the organisation or performance of the research, for example a pharmaceutical company, academic hospital, scientific organisation or investigator. A party that provides funding for a study but does not commission it is not regarded as the sponsor, but referred to as a subsidising party.
SUSAR	Suspected Unexpected Serious Adverse Reaction
Th1	T helper cell type 1
Th2	T helper cell type 2
Treg	Regulatory T cell (CD4+CD25+CD127- or CD4+CD25+FoxP3+)
Tresp	Responder T cell (CD4+CD25-)
Wbp	Personal Data Protection Act (in Dutch: Wet Bescherming Persoonsgevens)
WMO	Medical Research Involving Human Subjects Act (Wet Medisch-wetenschappelijk Onderzoek met Mensen

SUMMARY

Rationale: In earlier studies, we observed a positive correlation between vitamin D status and the regulation of the T cell compartment in MS patients.

Objective: The goal of this study is to assess whether supplementation of high doses vitamin D_3 results in a promotion of T cell regulation. Meanwhile, the effectiveness in raising serum 25(OH)D levels, as well as the safety of this intervention with regard to calcium homeostasis will be monitored.

Study design: This is a cohort-study.

Study population: A total number of 15 participants will be included. These are interferon beta 1a or 1b-treated relapsing remitting MS patients older than 18 years, with a disease course shorter than 6 years. They have no disorders of the vitamin D hormonal system, or disorders which augment their susceptibility for hypercalcemia. Additionally, pregnant women will not take part in this study.

Intervention (if applicable): Patients will be supplemented with 500 μg vitamin D_3/day in the form of Vigantol® (Merck™) solution for 12 weeks.

Main study parameters/endpoints: The effect of vitamin D supplementation on the T-cell compartment and in particular Treg function will be assessed. Secondly, serum 25(OH)D values, serum total calcium levels and kidney- and liver function will be monitored throughout the study.

Nature and extent of the burden and risks associated with participation, benefit and group relatedness: Patients have to visit the hospital 6 times, donate blood 6 times, and have to use the vitamin D_3 solution every day. The potential risk of hypervitaminosis D is hypercalcemia. Previous studies with high-dose vitamin D supplementation in MS found no hypercalcemia. Additionally, we exclude all patients which are potentially more sensitive for hypervitaminosis D and hypercalcemia. This study will provide valuable evidence on the effect of vitamin D on the T-cell compartment of MS patients.

1. INTRODUCTION AND RATIONALE

We are dependent upon our environment for acquiring vitamin D. Although a minor part is acquired by diet, most vitamin D is synthesised by exposure of the skin to sunlight. In the serum, vitamin D is predominantly present as its biologically inactive metabolite 25-hydroxyvitamin D (25(OH)D) (Hollis et al. 2007), and this metabolite is widely accepted to be the best indicator of vitamin D status (Hollis, 1996). Upon signals from calcium metabolism, 25(OH)D is further hydroxylised by the kidneys towards 1,25-dihydroxyvitamin D (1,25$(OH)_2$D), the biologically active vitamin D metabolite. However other cell types, like T cells (Correale et al. 2009) and Macrophages (Smolders et al., 2008a), can also activate vitamin D. The balance between the different vitamin D metabolites in the serum and presumably also in the tissues is dependent upon several factors, including genetic polymorphisms of the vitamin D receptor (Smolders et al. 2009a; Smolders et al. 2009b).

An impaired vitamin D status leads to a decompensation of calcium metabolism, resulting in reduced serum calcium levels and bone mass density. 25(OH)D levels below 75 nmol/L have been proposed to be insufficient for maintaining a healthy calcium metabolism (Dawson-Hughes et al. 2005). Recent studies attribute additional roles to vitamin D in body homeostasis, especially in immune regulation. Impaired levels of vitamin D have been associated with several autoimmune diseases, including inflammatory bowel disease, systemic lupus erythematosus and multiple sclerosis (MS). We are interested in the latter association.

Low serum levels of 25(OH)D in adolescence and limited sun exposure in childhood have been associated with an increased incidence of MS (Munger et al. 2007). In MS patients, low 25(OH)D levels have been associated with increased levels of disability in the entire MS population, and with a lower chance to remain relapse free in relapsing remitting MS (RRMS) patients with a short disease duration (Smolders et al. 2008a). In the pathophysiology of MS, auto-reactive T helper type 1 (Th1) and Th17 cells and a compromised regulatory T cell (Treg) function play an essential role. *In vitro* research suggest that vitamin D skews the immune response from an inflammatory Th1/ Th17 towards an anti-inflammatory Th2/ Treg phenotype (Smolders 2008b). Interestingly, preliminary data from our study 'Vitamin D and the T cell compartment in multiple sclerosis' (NL 22666.096.07) shows that serum 25(OH)D levels correlate negatively with the IFN-γ/IL-4 ratio, and with a better function of Treg in RRMS patients with a short disease course. These findings indicate that vitamin D modulates the T cell response towards a less inflammatory phenotype in MS patients *in vivo*. Interestingly, treatment with vitamin D can prevent and even cure animals in an experimental model of MS (Smolders et al. 2008a). Therefore, we would like to assess in the present study whether elevation of 25(OH)D levels by vitamin D_3 supplementation results in the anticipated shift within the T cell compartment.

The serum 25(OH)D level which should be achieved for an optimal immune regulation is uncertain. Based on measurements of 25(OH)D levels in primates in Africa and coast guards in Israel, it has been suggested that our immune system has been designed to be exposed to 25(OH)D levels exceeding 150 nmol/L (Vieth, 2005). Recently, it was shown that the correlation between vitamin D and

25(OH)D in the serum reaches a plateau phase at 25(OH)D levels of 100 nmol/L (Hollis et al. 2007), indicating that the formation of 25(OH)D is sated at levels exceeding 100 nmol/L. Furthermore, adolescents with serum 25(OH)D levels >100 nmol/L were found to have a reduced risk for developing MS in later life, compared to adolescents with the lowest serum 25(OH)D values (Munger et al., 2006). With vitamin D therapy in this study, we aim for 25(OH)D levels exceeding these concentrations. In our recent study 'Vitamin D and the T cell compartment in multiple sclerosis' (NL 22666.096.07), 50% of RRMS patients with a short disease course had 25(OH)D levels <50 nmol/L, and only 3 patients exceeded the 100nmol/L. Interestingly, these three patients had all an optimal Treg function. These figures suggest that, when supplementing vitamin D in an attempt to modulate the immune response, vitamin D levels should exceed at least 100-150 nmol/L.

To determine the dose of vitamin D_3 to reach these levels, one has to take safety into account. The only potential side effect of vitamin D supplementation, is decompensation of calcium metabolism and elevation of serum calcium values (hypercalcemia). Hypercalcemia has been rarely described in vitamin D_3 supplemented subjects (Hathcock et al. 2007). The issue of safety will be specifically addressed in section 5.3. Two studies did assess the effect of vitamin D_3 supplementation on 25(OH)D levels in MS patients. One study provided in weekly boluses the equivalent of 1000 µg/d, and reached a mean level of 325 nmol/L (min-max range 200-800 nmol/L) (Kimball et al. 2008). Another study supplemented patients with 250 µg/d for one year, reaching mean 25(OH)D levels of 413 nmol/L (min-max range 66 – 729) (Burton et al., 2008). Since we want to elevate 25(OH)D levels in all our patients at least above 100-150 nmol/L, we want to supplement our cohort with 500 µg/d. In several studies with vitamin D, it takes about 3 months for serum 25(OH)D levels to reach a new plateau phase. Therefore, subjects will receive 12 weeks supplementation.

To summarize, we would like to supplement a small cohort of RRMS patients, treated with interferon beta 1a or 1b, with a short disease course (<5 years) with high doses of vitamin D_3 in an open label design. The main goal is to determine whether this dose is effective in modulating the T cell compartment. The primary outcome measure will be improvement of Treg function in proliferation suppression assays. Furthermore, specific safety issues will be assessed. Does an acute start with high doses vitamin D_3 provoke side effects? Is calcium metabolism well regulated? Are serum 25(OH)D also effectively elevated in these patients using interferon beta 1a or 1b? The latter question is highly relevant, since therapy with Interferon Beta 1a and 1b specifically inhibits the superfamily of cytochrome P450 enzymes, to which the vitamin D hydroxylating enzymes also belong.

In MS,T cell regulation has been shown to be impaired. At present, disease modulating drugs in MS, like Beta Interferons, promote T cell regulation. The positive correlation between vitamin D status and T cell regulation that we observed in our previous study suggests that elevation of vitamin D status might result in promotion of T cell regulation. It is tempting to speculate that this promotion of T cell regulation might result in a subsequent amelioration of MS. Therefore, this study will be an important step towards assessment of vitamin D therapy in MS.

2. OBJECTIVES

Primary Objective:

- Does vitamin D_3 supplementation with a dose of 500 μg/ day for 12 weeks result in an improved regulatory T cell function (i.e. reduction of ED50 in the *in vitro* assay) in relapsing remitting MS patients treated with interferon beta?

Secondary Objective(s):

- Does vitamin D_3 supplementation with a dose of 500 μg/ day for 12 weeks result in a shift of the T cell compartment from a Th1 to a Th2 response in relapsing remitting MS patients treated with interferon beta?

- Is supplementation of vitamin D_3 with a dose of 500 μg/ day for 12 weeks effective in raising 25(OH)D levels above 100 nmol/L in relapsing remitting MS patients treated with interferon beta?

- Is supplementation of vitamin D_3 with a dose of 500 μg/ day for 12 weeks safe and tolerable in relapsing remitting MS patients treated with interferon beta?

3. STUDY DESIGN

The study will be a cohort study in 15 RRMS patients with a short disease course who are treated with interferon beta 1a or 1b. In this single study arm, vitamin D will be supplemented as a commercially available oil-based vitamin D_3 solution (Vigantol®, Merck^{TM}) at a dose of 500 µg/ day for 12 weeks. Four weeks after termination of vitamin D_3 therapy, biochemical markers will be checked to ensure normalization. See the flow-chart below (time indicated in weeks). This will be discussed in detail later.

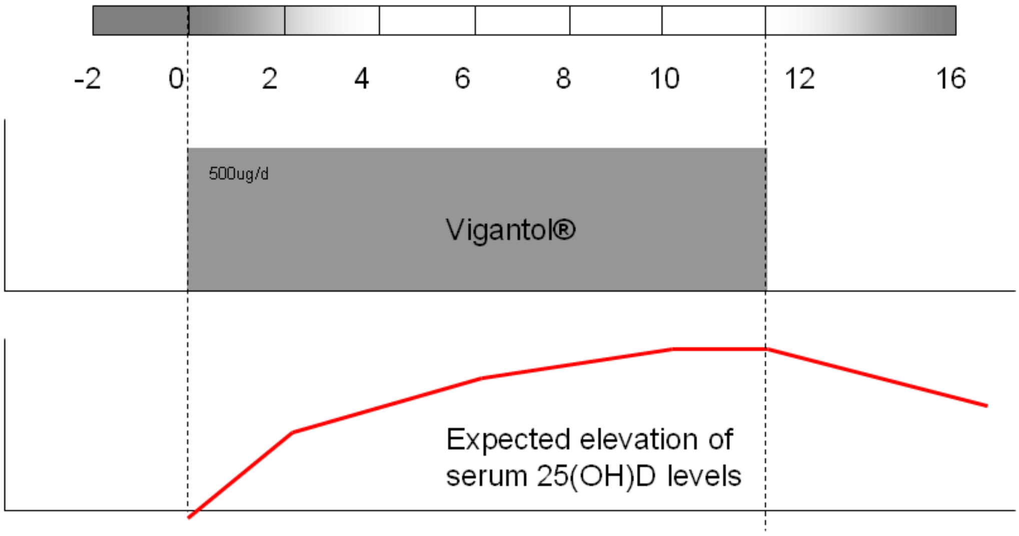

4. STUDY POPULATION

4.1 Population (base)

The patient cohort of the study will be collected from MS patients visiting our outpatient clinic, and from the patients which were included for the study 'Vitamin D and the T cell compartment in multiple sclerosis' (NL 22666.096.07) and indicated on their informed consent form that they would like to be invited to take part in future studies on vitamin D and MS (about 25 Beta Interferon users).

All subjects who are potentially more sensitive to develop hypercalcemia or complications from an eventual hypercalcemia (according to Hathcock et al. 2007) will be excluded to take part in this study (see exclusion criteria). A standard case report form (CRF) will be used to assess these issues at inclusion, with the additional performance of an ECG.

TOESTEMMINGSVERKLARING

Voor deelname aan het wetenschappelijk onderzoek:

Vitamine D status en het T cel compartiment in multipele sclerose

Ik ben over het onderzoek geïnformeerd. Ik heb de schriftelijke informatie (versie Maart 2008) gelezen. Ik ben in de gelegenheid gesteld om vragen over het onderzoek te stellen. Ik heb over mijn deelname aan het onderzoek kunnen nadenken. Ik heb het recht mijn toestemming op ieder moment weer in te trekken zonder dat ik daarvoor een reden hoef op te geven.

Ik stem toe met deelname aan het onderzoek, en geef hierbij tevens toestemming voor het gebruik van mijn medische- en onderzoeksgegevens, zoals omschreven in de informatiebrief (versie Maart 2008).

Mijn huisarts mag wel / niet (A.U.B. Uw keuze aangeven) geïnformeerd worden over mijn deelname aan dit onderzoek.

Over de resultaten van dit onderzoek (zoals die op mij van toepassing zijn) wens ik wel / niet (A.U.B. Uw keuze aangeven) geïnformeerd te worden.

Ik wens wel / niet (A.U.B. Uw keuze aangeven) benaderd te worden voor deelname aan eventueel vervolgonderzoek.

Naam :
Geboortedatum :

Handtekening : Datum :

(Vanaf hier in te vullen door onderzoeker)

Ondergetekende, verantwoordelijk onderzoeker, verklaart dat de hierboven genoemde persoon zowel schriftelijk als mondeling over het bovengemelde onderzoek is geïnformeerd. Hij/ zij verklaart tevens dat een voortijdige beëindiging van deelname door bovengenoemde persoon, van geen enkele invloed zal zijn op de zorg die hem of haar toekomt.

Naam :
Functie :

Handtekening : Datum :

4.2 Inclusion criteria

Inclusion criteria for the patient cohort are:

- Relapsing Remitting MS clinical phenotype (Revised McDonald criteria 2005)
- Disease duration from MS onset (first symptoms attributable to MS) < 6 years.
- At start of study > 6 weeks in clinical remission of disease
- Use of Interferon beta (1a or 1b) as immune modulation.
- Age > 18 years.

4.3 Exclusion criteria

Exclusion criteria are:

- Progressive phenotype of MS
- Relapse (treated or untreated with steroids) < 6 weeks before start study.
- Abnormalities of vitamin D hormonal system other than low dietary intake or limited sun exposure (i.e. Malabsorption, Cirrhosis, Nephrotic syndrome, Hyperthyreoidism, Renal failure, Rickets, hypoparathyreoidism, known malignancy, granulomatous disorders (TB, Sarcoidosis, Silicosis), and lymphomas)
- Intake of drugs that influence vitamin D homeostasis other than corticosteroids (i.e. Orlistat and anticonvulsants).

- Conditions associated with an increased susceptibility for hypercalcemia (know arrhythmia or heart disease, treatment with digitalis or hydrochlorothiazide, and those who suffer from nephrolithiasis).
- Frequent use of tanning bed (> 1 time/week).
- Alcohol or drug abuse
- Pregnancy or the intention to become pregnant during the study period.

4.4 Sample size calculation

We use the preliminary data of the study ‘Vitamin D and the T cell compartment in multiple sclerosis’ (NL 22666.096.07) for the power calculation. The inserted graph shows the relationship between Treg function and serum 25(OH)D levels in our previous study. In our suppression assay, we cultured Treg and responder T cells (Tresp) in different ratios (Treg/ Tresp 1:4 = 0.25). De Treg/Tresp ratio at which 50% suppression of Tresp proliferation was achieved, was set as ED50. We found that ED50 correlated negatively with serum 25(OH)D values; i.e. that Tregs were more able to suppress T cell proliferation in patients with high 25(OH)D levels.

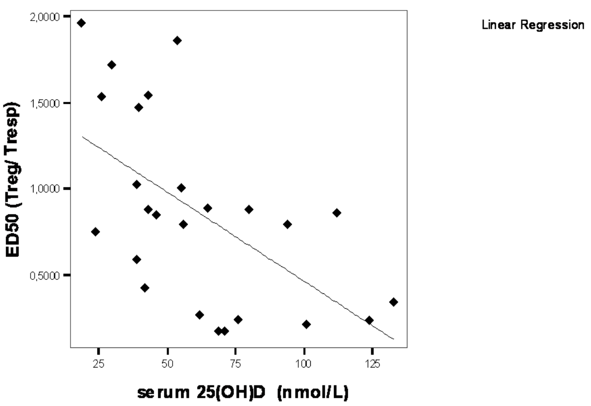

In the cohort with serum 25(OH)D levels below 100 nmol/L, mean ED50 was 1.0413 (SD 0.5503). This means that in the proliferation suppression assay, the Treg/Tresp ratio had to be approximately 1:1 to achieve 50% suppression of Tresp proliferation compared with the proliferation of stimulated Treg alone. In the study which has been conducted with vitamin D therapy at a dose of 250 µg/d, 25(OH)D levels were elevated to a mean of 413 nmol/L (Burton et al). Therefore we can expect (if the correlation we found can be modified by vitamin D therapy) the Treg/Tresp ratio to be very low. The culture condition with the lowest Treg/Tresp concentration that we use is 1 Treg on 4 Tresp (corresponding ED50: 0.25). When we take this value as a target for our primary endpoint, the expected difference is 1.0413-0.25 = 0,7912. Together with an power of 80% and alpha of 0.05, this results in the following formula for 2-sided significance:

$N = (2\alpha + 2\beta)^2 \text{ x } (2 \text{ x } SD^2)/ (\text{exp difference})^2 = (1{,}96 + 0{,}84)^2 \text{ x } (2 \text{ x } 0{,}5503)^2/ (0{,}7912)^2 = 7{,}84 \text{ x}1{,}93502$ ≈ 15 patients needed.

5. TREATMENT OF SUBJECTS

5.1 Investigational product/treatment

The subjects will be supplemented with Vigantol® oil. This is a commercially available (Merck™) oil-based solution of vitamin D_3 in a concentration of 500 µg/ mL. We purchase the product directly from the company, which ensures quality control. The patients will have to take 1 ml of the solution at breakfast. The pipette which is enclosed with the bottles can be used to dose the drops. The patients has to drop 40 drops on a spoon. The product is tasteless and can be taken with any other food product.
Vigantol® is registered as a medicine in Germany to prevent vitamin D deficiency in healthy people without resorbtion disorders, and to supplement vitamin D in vitamin D deficiency (see enclosed 'fachinformation' or product information (SPC)). In the Netherlands, vitamin D is not registered as a medicinal product and is regarded as a supplement (Dutch: voedingssupplement). It is in this study not used to treat any disease, but merely to additionally support the daily intake of participants by food and sun exposition to a higher level.

5.2 Use of co-intervention

Patients who take part in this study are treated with interferon beta 1a (Rebif®) or Interferon Beta 1b (Betaferon® or Avonex®) and have to continue this treatment. When taking multivitamin supplements, they can be continued when the incorporated amount of vitamin D does not exceed 5 µg/day. Excessive eating of products which contains lots of calcium (dairy products) or vitamin D (fat fish and cod liver oil, in lesser amounts meat, eggs, dairy products, avocado) should be discouraged. As part of the routine visits at week 1, 6 and 12, the calcium and vitamin D intake will be inventoried. Also, the amount of sunlight exposure and recent sun-vacations will be assessed. In case of a relapse of MS during the study period, this can be normally treated with corticosteroids.

5.3 Escape medication

There is no escape medication for vitamin D. There is no evidence-based cut-off value for hypervitaminosis D. Vitamin D levels are too high when calcium metabolism starts to decompensate and serum levels of calcium rise to pathological levels (serum total calcium >2.60 mmol/L). In adults with a normal parathyroid function, this only happens at daily vitamin D intakes of 1.000–2500 µg/ day (40.000–100.000 IU) (See SPC Vigantol®). We supplement our patients with only 500 µg/ day (20.000 IU). Although whole-day-long total-body sun-exposure

could theoretically provide the equivalent of 250 µg vitamin D intake, the total intake stays still well below the intake needed to develop hypercalcemia.
Several studies support the safety of supplying high doses of vitamin D to MS patients. Vigantol® has been used to supplement children with vitamin D (Maalouf J, et al. 2008). There were no side effects reported. One study supplemented to 12 MS patients the equivalent of 1.000 µg/d (40.000 IU) in weekly boluses of 7.000 µg (280.000 IU) for 28 weeks. The mean serum 25(OH)D level at study termination was 325 nmol/L (min-max range 200-800 nmol/L) (Kimball et al. 2008). Another study supplemented to 24 MS patients 250 µg/d (5.000 IU) for 52 weeks. The maximum mean 25(OH)D was 413 nmol/L (min-max range 66-720 nmol/L) (Burton et al. 2008). In both studies, no decompensation of calcium metabolism (serum total calcium and urine Ca^{2+}/ creatinine rate) was observed and no side effects were reported.

Therefore, the risk of our intervention to decompensate calcium metabolism and to elicit a hypercalcemia is very low. However, to supply vitamin D as safe as possible, we will follow our patients intensively. The first 6 weeks of the study, we do regular blood tests and instruct the patients to contact their neurologist if any side-effect occur. Potential side effects of hypercalcemia include: nausea, vomiting, diarrhoea, constipation, anorexia, apathy, pain in head, pain in muscles or joints, muscle weakness, sleepiness, uraemia (tiredness, decreased libido), polydypsia (excessive thirst), or polyuria (passage of large volumes of urine) (see SPC Vigantol®). When patients report these complaints, serum total calcium levels will be checked additionally.

In the unlikely case that hypercalcemia occurs in the current regimen, the vitamin D supplementation has to be stopped. Normalisation of serum calcium will be fastened by applying calcium-free nutrition, and promoting an excessive fluid intake. Eventually, a forced diuresis by furosemide can be applied. In case of a very severe hypercalcemia, subjects with a normal kidney-function can be given isotonic NaCl-solution (3-6 l/24h) to wash out the calcium. In case of oligo- or anuria, hemodialysis might be indicated.

6. METHODS

6.1 Study parameters/endpoints

6.1.1 Main study parameter/endpoint

The main study endpoint is the function of regulatory T cells ($CD4^{+}CD25^{+}CD127^{-}$) in the T cell compartment. This will be assessed by testing their capability to inhibit CD3-driven Tresp ($CD4^{+}CD25^{-}$) cell proliferation in culture.

6.1.2 Secondary study parameters/endpoints (if applicable)

Several other T cell characteristics of the T cell compartment will also be investigated. The number of regulatory T cells in the circulation, as well as the percentages of individual T helper cell subsets within the $CD4^{+}$ T cell compartment will be determined by flow-cytometry.
Several parameters will be assessed to investigate the safety of vitamin D_3 supplementation. These are:

- Plasma levels of total calcium, albumin and parathyroid hormone (PTH) to assess a potential decompensation of calcium metabolism.
- Plasma levels of phosphate, potassium, sodium, creatinine and urea to assess a potential decompensation of kidney function.
- Plasma levels of aspartate aminotransferase (AST), alanine aminotransferase (ALT), alkaline phosphatase (AP) to assess a potential decompensation of liver function.

The elevation of serum 25(OH)D and $1,25(OH)_2D$ levels will be measured to assess the effectiveness of the intervention.

6.2 Randomisation, blinding and treatment allocation

This pilot study has only one arm. All participants receive the same intervention and are not randomised.

6.3 Study procedures

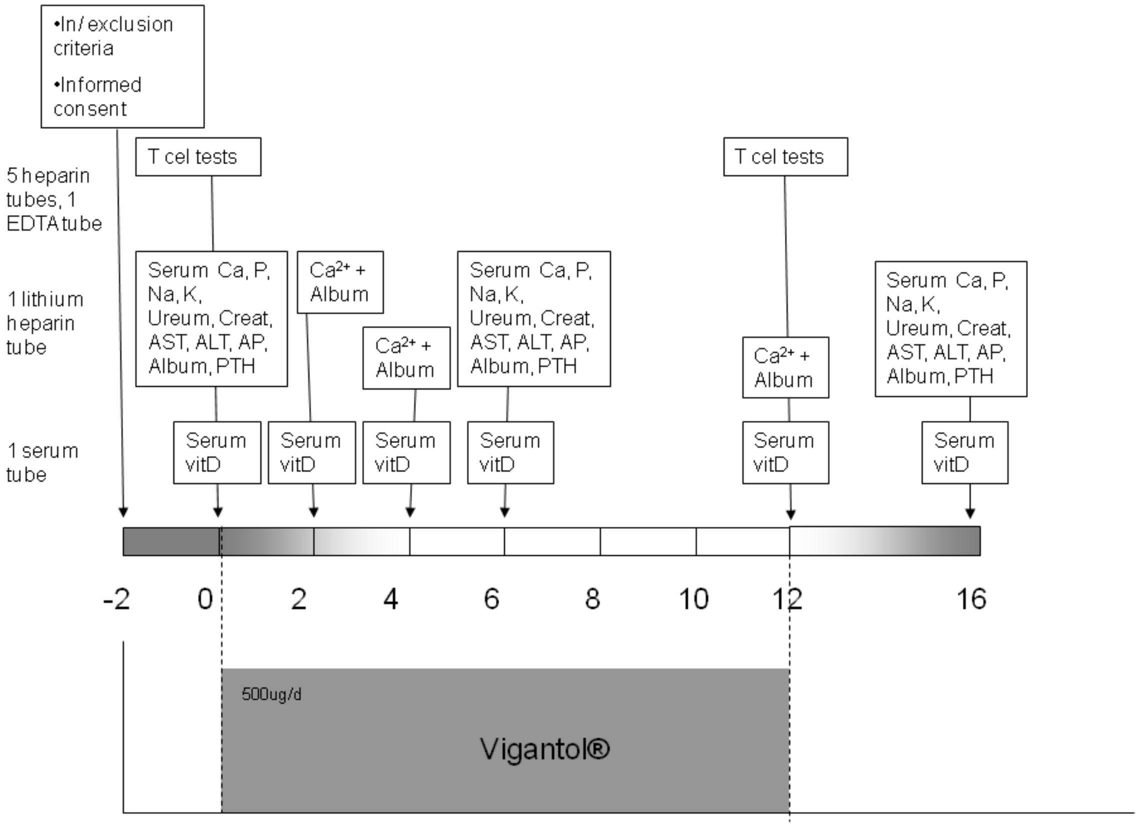

The study is outlined in the figure presented above (time in weeks). At inclusion, patients will be assessed with a standard CRF (see attachment), to check the in- and exclusion criteria. If the patient matches the inclusion criteria, and is willing to sign the informed consent form, he or she will be included in the study.

At the baseline visit, blood will be collected to assess baseline-values before intervention. Herefore, 8 tubes of blood will be drawn by an experienced research nurse. The subjects will receive the Vigantol® for the first 6 weeks of the study (5 flacons à 10 ml) and instructions on how to dose and use the pipette. Additionally, exposure to vitamin D by diet and sunlight exposure and exposure to calcium by diet will be assessed. The patient takes the following dose:

Week 1-6	500 μg/d	1.0 ml	40 drops	total 42.0 ml
			Total week 1-6	50.0 ml

Appointments will be made for the blood controls at week 2 (2 tubes), 4 (2 tubes) and 6 (2 tubes). When coming for a blood monster in the hospital, a short standardised contact with a research nurse will be arranged to assess side-effects and promote compliance. At the

control appointment at week 6 of the study, the amount of Vigantol left in the bottles will be checked, to assess compliance objectively. Dietary intake by calcium and vitamin D and sunlight exposure will be assessed. The remainder of the Vigantol to complete the study will be provided (5 flacons à 10 ml), and the control appointments at week 12 and 16 will be made.

Week 7-12	500 µg/d	1.0 ml	40 drops	total 42.0 ml
			Total week 7-12 50.0 ml	

At the appointment at week 12, the remainder of Vigantol in the bottles will again be assessed. Additionally, calcium and vitamin D intake will be assessed. At this time-point, a total of 7 tubes of blood will be collected to assess the T cell characteristics.

After completing the study protocol in week 12, patients will immediately stop vitamin D_3 therapy. A last control is at week 16 to check the biochemical markers and wellbeing of the patient. After this last control, patients are allowed to continue vitamin D supplementation, however it is advised to restrict to the doses indicated by the report of the Dutch Health Counsel (Health Counsel of the Netherlands, 2008), in which supplementation with 20 µg/ day is indicated for patients at risk for an impaired vitamin D status. For this purpose, commercially available vitamin D_3 tablets (For instance vitamin D_3, 2 pills of 10 µg, HEMA) can be advised. The patient information letter will encourage patients to get advice from their treating physician, general practitioner or dietary advisor.

All clinical chemical variables will be determined by the standard operating protocols of the Chemical and Haematological Laboratory of the Orbis Medical Center in Sittard. The vitamin D values will be determined by radioimmune assays following the standard operating protocols of the Chemical Laboratory of Maastricht University Medical Center in Maastricht. All vitamin D metabolite levels will be determined in the same run, using the same batch of radionucleotide.

The cellular assays are performed following the standard operating protocols of the experimental immunology laboratory of the Maastricht University Medical Center, according to the procedures used in study (NL 22666.096.07).
In short, peripheral blood mononuclear cells (PBMC's) will be isolated directly from heparinised blood by Ficoll gradient. Phenotypical analysis will be performed by intracellular and surface Flow Cytometry.

6.4 Withdrawal of individual subjects

Subjects can leave the study at any time for any reason if they wish to do so without any consequences. The investigator can decide to withdraw a subject from the study for urgent medical reasons.

6.4.1 Specific criteria for withdrawal (if applicable)

Subjects will be withdrawn from the study if any of the following events occur:

- The reference values for serum total calcium are 2.10–2.55 mmol/L. If the serum total calcium level exceeds 2.55 mmol/L, the patient has to stop vitamin D supplementation until the measurement has been repeated . If the serum calcium level repetitively exceeds 2.55 mmol/L, the patient is withdrawn from the study. If not, the patient can continue the study.
- If liver- or kidney-function worsens clinically significant at week 6 when compared to baseline values, the patient will be withdrawn from the study and has to stop vitamin D supplementation immediately.

6.5 Replacement of individual subjects after withdrawal

Withdrawn patients will be replaced by other patients matching the inclusion criteria.

6.6 Follow-up of subjects withdrawn from treatment

Subjects withdrawn from treatment, will have their safety measurements repeated at 2 weeks and 8 weeks after withdrawal from the study, to assess if their calcium levels normalise. The $T^1/_2$ of 25(OH)D is approximately 30 days.

6.7 Premature termination of the study

The study will be terminated when more than 2 patients of the cohort experience a hypercalcaemia, or severe functioning of liver and/or kidney function as described in section 6.4.1.. All patients have to stop supplementation and will be followed as described in section 6.6.

7. SAFETY REPORTING

7.1 Section 10 WMO event

In accordance to section 10, subsection 1, of the WMO, the investigator will inform the subjects and the reviewing accredited METC if anything occurs, on the basis of which it appears that the disadvantages of participation may be significantly greater than was foreseen in the research proposal. The study will be suspended pending further review by the accredited METC, except insofar as suspension would jeopardise the subjects' health. The investigator will take care that all subjects are kept informed.

7.2 Adverse and serious adverse events

Adverse events are defined as any undesirable experience occurring to a subject during a clinical trial, whether or not considered related to the supplementation of vitamin D_3 by Vigantol® oil. All adverse events reported spontaneously by the subject or observed by the investigator or his staff will be recorded.

A serious adverse event is any untoward medical occurrence or effect that at any dose results in death;

- is life threatening (at the time of the event);
- requires hospitalisation or prolongation of existing inpatients' hospitalisation;
- results in persistent or significant disability or incapacity;
- is a congenital anomaly or birth defect;
- is a new event of the trial likely to affect the safety of the subjects, such as an unexpected outcome of an adverse reaction, lack of efficacy of an IMP used for the treatment of a life threatening disease, major safety finding from a newly completed animal study, etc.

All SAEs will be reported to the accredited METC that approved the protocol, according to the requirements of that METC.

7.2.1 Suspected unexpected serious adverse reactions (SUSAR)

Adverse reactions are all untoward and unintended responses to an investigational product related to any dose administered.

Unexpected adverse reactions are adverse reactions, of which the nature, or severity, is not consistent with the applicable product information in the Summary of Product Characteristics (SPC) of Vigantol® as provided by the manufacturer.

The sponsor will report expedited the following SUSARs to the METC:

- SUSARs that have arisen in the clinical trial that was assessed by the METC;
- SUSARs that have arisen in other clinical trial with Vigantol®, and that could have consequences for the safety of the subjects involved in the clinical trial that was assessed by the METC.

The remaining SUSARs are recorded in an overview list (line-listing) that will be submitted once every half year to the METC. This line-listing provides an overview of all SUSARs from the study medicine, accompanied by a brief report highlighting the main points of concern.

The expedited reporting will occur not later than 15 days after the sponsor has first knowledge of the adverse reactions. For fatal or life threatening cases the term will be maximal 7 days for a preliminary report with another 8 days for completion of the report.

7.2.2 Annual safety report

In addition to the expedited reporting of SUSARs, the sponsor will submit, once a year throughout the clinical trial, a safety report to the accredited METC.

This safety report consists of:

- a list of all suspected (unexpected or expected) serious adverse reactions, along with an aggregated summary table of all reported serious adverse reactions, ordered by organ system, per study;
- a report concerning the safety of the subjects, consisting of a complete safety analysis and an evaluation of the balance between the efficacy and the harmfulness of the substance under investigation.

7.3 Follow-up of adverse events

All adverse events will be followed until they have abated, or until a stable situation has been reached. Depending on the event, follow up may require additional tests or medical procedures as indicated, and/or referral to the general physician or a medical specialist.

8. STATISTICAL ANALYSIS

8.1 Descriptive statistics

All data will be analysed descriptively. The biochemical (vitamin D metabolite levels, calcium parameters, liver function tests, and kidney function tests) and immunological data (T cell subset percentages, percentage of Treg suppression) will be presented as continuous variables. No cut-off points will be used for any value. The demographical data will also be presented either as continuous or as categorical variables as appropriate. Of continuous data, medians and minimum-maximum ranges will be provided. Of categorical data, counts and corresponding percentages will be provided.

8.2 Univariate analysis

The effect of the intervention on T cell characteristics (primary outcome measures) will be expressed as the difference between the respective continuous variables (ED50, T cell subsets or Th1/Th2 ratios) before and after treatment. The significance of this difference will be tested with a paired non-parametrical test (Wilcoxon ranking test). The same holds for the effect of the intervention on safety parameters and vitamin D metabolite values.

8.3 Multivariate analysis

No multivariate analysis will be performed.

9. ETHICAL CONSIDERATIONS

9.1 Regulation statement

This study will be conducted according to the principles of the Declaration of Helsinki (version DoH/Oct2008, as accorded by the 59th WMA general assembly in Seoul, October 2008) and in accordance with the Dutch Medical Research Involving Human Subjects Act (WMO).

9.2 Recruitment and consent

The patients will be primarily included from the population which were included for the study 'Vitamin D and the T cell compartment in multiple sclerosis' (NL 22666.096.07). Those patients all agreed to be approached for further studies. Additionally, patients from outside this cohort matching the inclusion criteria will be approached. Patients who meet the inclusion criteria will be contacted by a research nurse. When interested, a patient brochure will be send. In this brochure, telephone numbers and e-mail addresses of the researcher and independent physician are provided. If the patient agrees to take part in the study, and appointment will be made to further check the inclusion criteria, answer eventual questions, and to sign the informed consent form.

9.3 Objection by minors or incapacitated subjects (if applicable)

No minors and/or incapacitated adults will be included in this study.

9.4 Benefits and risks assessment, group relatedness

Several safety studies provided evidence that supplementation of MS patients with high dose vitamin D is safe (section 5.3). Nevertheless, patients have to take the Vigantol® each day, which requires both time and commitment from our patients. Additionally, patients have to donate blood 6 times. This is as less often as possible; safety controls will be performed by assessing the serum calcium levels. The risks of a blood donation are a temporary vasovagal reaction or a local haematoma at the puncture spot.

There is at present no direct therapeutic effect of this regimen to be expected in participants. However, an effect of this intervention on the T cell compartment would provide an essential argument to investigate a therapeutic effect of vitamin D supplementation on MS.

9.5 Compensation for injury

The sponsor/investigator has a liability insurance which is in accordance with article 7, subsection 6 of the WMO. The insurance company is 'Vereniging Voor Arts en Auto' and the polis number 1150888 ('Orbis Medisch en zorgconcern').

The sponsor (also) has an insurance which is in accordance with the legal requirements in the Netherlands (Article 7 WMO and the Measure regarding Compulsory Insurance for Clinical Research in Humans of 23th June 2003). The insurance company is MediRisk B.A. (Orteliuslaan 750, Po-box 8409, 3503RK, Utrecht). The polis is on the name of 'Orbis Medisch en Zorgconcern' and has the number MR017/020924/WMO. This insurance provides cover for damage to research subjects through injury or death caused by the study.

1. € 450.000,-- (i.e. four hundred and fifty thousand Euro) for death or injury for each subject who participates in the Research;
2. € 3.500.000,-- (i.e. three million five hundred thousand Euro) for death or injury for all subjects who participate in the Research;
3. € 5.000.000,-- (i.e. five million Euro) for the total damage incurred by the organisation for all damage disclosed by scientific research for the Sponsor as 'verrichter' in the meaning of said Act in each year of insurance coverage.

The insurance applies to the damage that becomes apparent during the study or within 4 years after the end of the study.

9.6 Incentives (if applicable)

Subject will receive no incentives for participating in the study. Travel costs will be compensated with the standard tariff of € 0.19 /km as measured by an electronic route planner.

10. ADMINISTRATIVE ASPECTS AND PUBLICATION

10.1 Handling and storage of data and documents

Data will be stored for each patient in one single master-file. After completing the informed consent form, patient will receive a code under which their data will be stored in the master file. Patients will receive a code at order of inclusion. Coding will start at 201, 202, 203, etc. Only the principal investigator has access to the informed consent forms and key to the code. Material which is stored (serum, plasma) will be labelled with the research code of the patient and will be destroyed at the end of the study.

10.2 Amendments

A 'substantial amendment' is defined as an amendment to the terms of the METC application, or to the protocol or any other supporting documentation, that is likely to affect to a significant degree:

- the safety or physical or mental integrity of the subjects of the study;
- the scientific value of the study;
- the conduct or management of the study; or
- the quality or safety of any intervention used in the study.

All substantial amendments will be notified to the METC.

Non-substantial amendments will not be notified to the accredited METC, but will be recorded and filed by the sponsor.

10.3 Annual progress report

The sponsor/investigator will submit a summary of the progress of the trial to the accredited METC once a year. Information will be provided on the date of inclusion of the first subject, numbers of subjects included and numbers of subjects that have completed the trial, serious adverse events/ serious adverse reactions, other problems, and amendments.

10.4 End of study report

The investigator will notify the accredited METC of the end of the study within a period of 8 weeks. The end of the study is defined as the last biochemical test performed on the last patients material.

In case the study is ended prematurely, the investigator will notify the accredited METC, including the reasons for the premature termination.

Within one year after the end of the study, the investigator/sponsor will submit a final study report with the results of the study, including any publications/abstracts of the study, to the accredited METC.

10.5 Public disclosure and publication policy

The results of this study, regardless their nature, will be published in a scientific paper, and/ or be presented at a scientific congress.

REFERENCES

- Burton J, Kimball S, Vieth R, Bar-Or A, Dosch H, Thibault L, Kilborn S, D'Souza C, Cheung R, Urseli M, O'connor P. A Phase I/II dose-escalation trial of oral vitamin D_3 with calcium supplementation in patients with multiple sclerosis. *Mult Scler* 2008; **14**: P20.
- CorrealeJ, Ysrraelit M, Gaitán M. Immunomodulatory effects of vitamin D in multiple sclerosis. *Brain* 2009; (published online): 2–15.
- Dawson-Hughes B, Heaney R, Holick M, Lips P, Meunier P, Vieth R. Estimates of optimal vitamin D status. *Osteoporos Int* 2005; **16**: 713–716.
- Health Counsil of the Netherlands. Towards an adequate intake of vitamin D. The Hague: Health Counsil of the Netherlands, 2008; publication number 2008/15.
- Hathcock J, Shao A, Vieth R, Heany R. Risk assessment of vitamin D. *Am J Clin Nutr* 2007; **85**: 6–18.
- Hollis B. Assessment of vitamin D nutritional and hormonal status: what to measure and how to do it. *Calcif Tissue Int* 1996; **58**: 4–5.
- Hollis B, Wagner C, Drezner M, Binkley N. Circulating vitamin D_3 and 25-hydroxyvitamin D in humans: an important tool to define adequate nutritional vitamin D status. *J Steroid Biochem Mol Biol* 2007; **103**: 631–634.
- Kimball S, Ursell M, O'Connor P, Vieth R. Safety of vitamin D_3 in adults with multiple sclerosis. *Am J Clin Nutr* 2007; **86**: 645–651
- Maalouf J, Nabulsi M, Vieth R, Kimball S, El-Rassi R, Mahfoud Z, El-Hajj Fuleihan G. Short- and long-term safety of weekly high-dose vitamin D_3 supplementation in school children. *J Clin Endocrinol Metab* 2008; **93**: 2693–701.
- Munger K, Levin L, Hollis B, Howard N, Ascherio A. Serum 25-hydroxyvitamin D levels and risk of multiple sclerosis. *JAMA* 2006; **296**: 2832–2838.
- Smolders J, Damoiseaux J, Menheere P, Hupperts R. Vitamin D as an immune modulator in multiple sclerosis, a review. *J Neuroimmunol* 2008; **194**: 7–17.
- Smolders J, Menheere P, Kessels A, Damoiseaux J, Hupperts R. Association of vitamin D metabolite levels with relapse rate and disability in multiple sclerosis. *Mult Scler* 2008; **14**: 1220–1224.
- Smolders J, Damoiseaux J, Menheere P, Cohen Tervaert JW, Hupperts R. Fok-I vitamin D receptor gene polymorphism (rs10735810) and vitamin D metabolism in multiple sclerosis. *J Neuroimmunol* 2009; **15**: 117–121.

- Smolders J, Damoiseaux J, Menheere P, Cohen Tervaert JW, Hupperts R. Association study on two vitamin D receptor polymorphisms and vitamin D metabolites in multiple sclerosis. *Ann N Y Acad Sci* 2009 *Article in press*.
- Vieth R. What is the optimal vitamin D status for health? *Prog Biophys Mol Biol* 2006; **92**: 26–32.
- Venken K, Hellings N, Hensen K, Rummens J, Medaer R, D'Hooge M. Hupperts R, Stinissen P. Secondary progressive in contrast to relapsing remitting multiple sclerosis patients show a normal CD4+CD25+ regulatory T-cell function and FokP3 expression. *J Neurosci Res* 2006; **83**: 1432–1446.
- Viglietta V, Baecher-Allan C, Weiner H, Hafler D. Loss of functional suppression by CD4+CD25+ regulatory T cells in patients with multiple sclerosis. *JEM* 2004; **199**: 971–979.
